# Creation of a Databank for Content of Antioxidants in Food Products by an Amperometric Method

**DOI:** 10.3390/molecules15107450

**Published:** 2010-10-22

**Authors:** Yakov I. Yashin, Boris V. Nemzer, Vadim Yu. Ryzhnev, Alexandr Ya. Yashin, Nina I. Chernousova, Polina A. Fedina

**Affiliations:** 1 Scientific Development & Production Center “Khimavtomatika”, Selskohozyaistvennaya 12a, 129226 Moscow, Russia; 2 VDF FutureCeuticals, Inc., 300 West 6th Street, Momence, IL 60954, USA

**Keywords:** water- and fat-soluble antioxidants, amperometric method, antioxidant therapy, databank

## Abstract

Oxidative stress, *i.e.* excessive content of reactionary, oxygen, and nitrogen compounds (ROAC), including free radicals, is one of the causes of various dangerous diseases as well as premature aging. The adverse effect of free radicals can be neutralized by antioxidants. In order to carry out antioxidant therapy, one needs to know the contents of antioxidants in food products. We have created the databank for the contents of antioxidants in 1,140 food products, beverages, *etc.* Apart from water-soluble antioxidants, fat-soluble antioxidants in dairy and fish products, cacao, chocolate, nuts *etc.* were determined for the first time using an amperometric method.

## 1. Introduction

Over the past decades various studies from different countries have confirmed that oxidative stress is one of the main causes of premature aging and many other diseases [1,2,3,4,5,6,7,8]. This means excessive contents of free oxygen radicals in biological liquids are in the human body such as superoxide anion, hydroxyl radical, perhydroxyl radical, *etc.* Free radicals are constantly formed, even with normal cell metabolism. Some data shows that 2% of total absorbed oxygen is transformed into free radicals. It is widely held that our body requires certain amounts of active forms of oxygen to help eliminate harmful bacteria, dying cells, *etc.* The antioxidant system of a healthy person provides for a normal and safe level of free radicals. However, if adverse factors (radioactive and UV radiation exposures, environmental pollution or low quality food products, stress, diseases, certain powerful drugs and treatment procedures, smoking, alcoholism, drug abuse, *etc.*) affect the body, the level of active oxygen forms (free radicals and peroxides) may also increase. Such forms may damage DNAs, proteins, lipids, carbohydrates as well as vascular walls, which results in the disorganization of normal processes in human bodies. To put it generally, it works as follows: adverse conditions → oxidative stress → oxidation of vital molecules → development of diseases or premature aging. 

Free radicals are especially reactive towards membrane lipids containing unsaturated bonds. As a result, the properties of cellular membranes are modified. The most active free radicals break bonds in the DNA molecule and injure the genetic apparatus of cells, which may lead to the development of oncological diseases. Upon oxidation, low-density lipoproteins can deposit onto the vascular walls and initiate the development of cardiovascular diseases. Presently, tens of diseases have been associated with oxidative stress.

To prevent oxidative stress, one should consume products with sufficient amounts of antioxidants, so that the harmful effect of free radicals is significantly diminished. The major natural antioxidants include flavonoids, aromatic oxyacids, vitamins C and E, carotenoids, and other compounds. The continual increase of the recommended amount of antioxidants should also be avoided, as some of them become pro-oxidants at elevated concentrations. The Russian Ministry of Health recommends consuming approximately 350 mg of different antioxidants per day. Sick and debilitated individuals, as well as those who are overworked and work in poor conditions are advised to consume around 1,000-1,300 mg. However, such recommendations specify only the general amount of antioxidants as, unfortunately, it is not yet possible to estimate the exact ratio for water- and fat-soluble antioxidants.

The aim of this study is a creation of database with results of antioxidant capacity of main food products: vegetables and greens, fruits, berries, juices, nuts, dairy products, seafood products, vegetable oils, honey, tea, coffee, different alcoholic beverages, *etc.*

## 2. Results and Discussion

Studies on evaluation of antioxidant activity (capacity) for various food products in different countries have been published [9,10,11,12,13]. Halvosen *et al*. [10] established the total content of antioxidants in fruits, berries, vegetables and other products determined by the Ferric Reducing Antioxidant Power (FRAP) method. The following berries contained the highest amount of antioxidants: rose hips, black currant, raspberry, bilberry, and cranberry. Fruits, berries and grains contribute 43.6%, 27.1%, and 11.7% of antioxidants in the diet of Norwegians, respectively. Vegetables contribute only 8% to the total content of plant antioxidants in the diet.

The USDA database of the flavonoids content in selected foods [14] provides the flavonoids composition in different foods and beverages, including raw and processed fruits, berries, vegetables, spices and such on. In particular, the following flavonoids and aromatic oxyacids have been indentified: hydroxy aromatic acids (gallic acid, dihydroxybenzoic acid, *p*-hydroxybenzoic acid), anthocyanidins (cyanidin, pelargonidin, peonidin, delphinidin, malvidin), anthocyanins, hydroxicoric acids (caffeic acid, chlorogenic acid, coumarinic acid, ferulic acid, syringic acid), flavanols (catechin, epicatechin), flavones (luteolin, apigenin). Only data generated by acceptable procedures defined as those which lead to good separation of flavonoids compounds (e.g. column chromatography, HPLC, capillary zone electrophoresis, micellar electrokinetic capillary chromatography) have been included in this database.

Thus, although over 5,000 flavonoids have been identified [15], only 50-100 of them are present in significant amounts in fruits, berries, vegetables, and other natural products and can be separated and identified by HPLC or LC/MS systems. Flavanones (isonaringin, naringenin, naringin, hesperetin, hesperidin, neohesperidin), flavanones (quercetin, auranetin), flavones (tangeretin, nobiletin) were indentified in oranges. 

Sakakibara *et al.* [16] classified over 100 of the most common antioxidants found in food products nd simultaneously separated by a Capcell pak C18 UG120 column (Shiseido Co., Ltd., Tokyo, Japan) and detected by a HPLC system equipped with a diode array detector. With such a column, the contents of antioxidants in food products may be expressly evaluated. 

In this work the total contents of water- and fat-soluble antioxidants in 1,140 food products, beverages, dietary supplements, herbal extracts, vitamins, medicines *etc.* have been estimated by an amperometric method using a TsvetYauza-01-AA (NPO Khimavtomatika Inc., Moscow, Russia) amperometric detector.

The following groups of products have been studied by this method: berries, fruits, vegetables, (potato separately), greens, dairy, meat and fish products, vegetable oils, cacao and chocolate, nuts and seeds, grains, sprouted grains, condiments and spices, tea, coffee, wine, beer, cognac, whisky, juices, beverages, dietary supplements, herbal extracts, medicinal-antioxidants, *etc.* This report includes the data of antioxidant capacity or antioxidants content in some food products and beverages (Table 1, Table 2, Table 3, Table 4, Table 5, Table 6, Table 7, Table 8, Table 9, Table 10, Table 11 and Table 12). Before each measurement, the device was calibrated according to standards; the correlation ratio for calibration curves was no less than 0.99. The used average value was based on 3-5 consecutive measurements; SD was less than 3%. Maximum permissible concentration (MPC) for major antioxidants was no higher than 10^-8^–10^-9^ g. Based on sample selection and preparation, the total error for the measurements was within ±10%. 

In Table 1 are presented data of total content of antioxidants (TCA) in berries and fruits. It is shown that lemon peel contains a significantly higher amount of antioxidants in comparison with the whole lemon or lemon pulp.

Table 2 contains TCA data for different vegetables and greens. We have separately studied more than 30 potato varieties provided by the Lorkh Institute of Potato Growing (Russia). TCA value for the best potato varieties was around 70 mg/100 g. According to the data presented at the Malta Polyphenols 2007: 4^th^ International Conference on Polyphenols Applications [17] among vegetables, in France, potatoes contribute the highest amount of antioxidants into the diet, and among fruits—Apples. 

Table 3 includes data of total content of fat soluble antioxidants (TCFA) in cheeses and dairy products. All samples have been purchased in grocery store in Moscow (Russia). In Table 4 are presented data of TCFA in seafood products and in Table 5 - TCFA results for vegetable oils. Gallic acid has been used as a standard for cheeses, dairy products and vegetable oils samples and quercetin – for seafood products. We did not find any data related to the contents of fat-soluble antioxidants in dairy or seafood products in the literature. There is a huge difference in TCFA results between salmon caviar samples – from 136 to 6 mg/100 g. 

**Table 1:** Total Content of Antioxidants (TCA) in Berries and Fruits (quercetin – standard).

No.	Name	TCA mg/100g
	*Berries*	
1	Chokeberry	800
2	Black currant	765
3	Black sour cherry	572
4	Hawthorn	570
5	Rose hip	530
6	Viburnum	495
7	Bilberry	406
8	Blueberry	335
9	Cranberry	270
10	Barberry	230
11	Black cherry	221
12	Wild strawberry	210
13	Red currant	200
14	Ashberry	200
15	Raspberry	171
16	Cowberry	143
17	Gooseberry	46
18	Black grapes	42
19	Sea buckthorn	40
	*Fruits*	
1	Lemon peel	285
2	Feijoa	243
3	Red orange	79
4	Lemon	74
5	Apple (Zhigulevskoye variety)	64
6	Pear	50
7	Kiwi	45
8	Apricot	38
9	Plum	30
10	Avocado	27
11	Nectarine	17
12	Peach	12
13	Banana	7

**Table 2:** Total Content of Antioxidants (TCA) in Vegetables and Greens (quercetin – standard).

No.	Name	TCA mg/100g
	*Vegetables*	
1	Garlic	273
2	Sweet red pepper	245
3	Beet	217
4	Turnip	135
5	Red onion	117
6	Sweet yellow pepper	92
7	Yellow onion	88
8	Bulb onion	79
9	Red cabbage	76
10	White onion	75
11	White cabbage	69
12	Tomato	64
13	Carrot	64
14	Radish	62
15	Eggplant	54
16	Potato	43
17	Cucumber	22
	*Greens*	
1	Parsley	110
2	Watercress salad (leaves)	100
3	Celery leaves	100
4	Cilantro	90
5	Watercress salad (stems)	70
6	Green onion	50
7	Dill	30
8	Salad-premium	10
9	Celery stem	10

**Table 3:** Total Content of Fat-Soluble Antioxidants (TCFA) in Cheese & Other Dairy Products (standard - gallic acid).

No.	Name	TCFA mg/100g	Manufacturer
	*Cheeses*		
1	Suluguni cheese	50	JSC Giaginsky Diary Plant, Russia
2	Parmesan Goya cheese 50%	25	Molfino Hermanos S.A., Argentina
3	Djigas cheese (PARM.) 40%	21	Zemaitijos Pienas, Lithuania
4	Svalya cheese	13	Agroaspect, LLC, Russia
5	Gauda cheese 48%	12	JSC Valio, Finland
6	Rossiysky cheese	12	Uglich, Russia
	*Dairy Products*		
1	Activia yogurt, enriched with ActiRe-gularis bifido-bacterium with herbs 2.2%	75	Danon Industry, LLC, Russia
2	Miracle-yogurt “Strawberry+wild strawberry” 2.4%	75	Vimm-Bill-Damm, LLC, Russia
3	Lianozovskoye milk 3.2%	72	Vimm-Bill-Damm, LLC, Russia
4	Curdled milk 3.2%	66	CJSC Opolye Holding Company, Russia
5	Fromage frais cottage cheese 0%	54	Senoble France, France
6	Dolce Vita sour cream 20%	49	CJSC Ozeretsky Dairy Complex, Russia
7	Vologodskoye butter 82.5%	24	CJSC Ozeretsky Dairy Complex, Russia
8	Sour cream 20%	19	CJSC Opolye Holding Company, Russia
9	Countryside House drinking cream 10%	13	Vimm-Bill-Damm, LLC, Russia

**Table 4:** Total Content of Fat-Soluble Antioxidants (TCFA) in Seafood Products (standard – quercetin).

No.	Name	TCFA mg/100g	Manufacturer
1	Grainy salmon caviar	136	Sakhalin, Russia
2	Canned Baltic sprats in oil	88	Kreon, LLC, Svetly, Russia
3	Cooked cod (cans)	31	Espersen, Denmark
4	Fish oil	30	Russia
5	Grainy salmon caviar	24	JSC ICE Meridian, Russia
6	Grainy salmon caviar	22	CJSC Northeast Company, Ltd, Russia
7	Herring (filet in oil)	22	Vachyunay, LLC, Russia
8	Elite grainy salmon caviar	17	JSC Fish-Processing Plant No.1, Russia
9	Red grainy salmon caviar	12	CJSC ITA Northern Company, Russia
10	Grainy salmon caviar	6	JSC Fish-Processing Plant No.1, Russia

**Table 5:** Total Content of Fat-Soluble Antioxidants (TCFA) in Vegetable Oils (standard - gallic acid).

No.	Name	TCFA mg/100g	Manufacturer
1	Ethiopian black cumin oil	294	Egypt
2	CAROTINO palm-tree oil	198	Malaysia
3	Olive oil	127	Greece
4	Sea buckthorn oil	123	VitaOil, LLC, Russia
5	Wheat-germ oil	109	DecosT Series, NPKF DecosT, LLC
6	Shark oil	73	Kubanrybprom, LLC, Russia
7	Inca Gold amaranthine oil	62	DecosT Series, NPKF DecosT, LLC, Russia
8	Corn oil	45	Efko, LLC, Russia
9	Pine nut oil	34	Altay, LLC, Russia
10	Cucurbits oil	31	DecosT Series, NPKF DecosT, LLC, Russia
11	Mustard oil	22	Life Aromas Series, NPKF DecosT, LLC, Russia
12	Flax oil	18	Life Aromas Series, NPKF DecosT, LLC, Russia
13	Celery oil	18	CJSC SPA Europe-Biopharm
14	Unfiltered walnut oil (crumb)	16	Life Aromas Series, NPKF DecosT, LLC, Russia

Interesting data was obtained upon detection of both water- and fat-soluble antioxidants in cacao and chocolate samples (Table 6). Russian samples of cacao contain significant amounts of water- and fat-soluble antioxidants because the cacao oil is not extracted from raw cacao material. Per serving, in terms of the general level of antioxidant activity, cacao exceeds tea and red wine. Moreover, cacao is one of few products containing the entire set of required antioxidants: water-soluble antioxidants, fat-soluble antioxidants, and anthocyanins. Cacao may be considered one of the most balanced food products taking into account that cacao contains fats, proteins, carbohydrates, microelements, and vitamins. No wonder that cacao and chocolate are considered “food of the Gods.” Fat-soluble antioxidants in nuts and seeds (Table 7) are in an approximate correspondence with the published data. 

**Table 6:** Total Content of Fat-Soluble Antioxidants (TCFA) (standard - gallic acid) and Water-Soluble Antioxidants (TCA) in Cacao, Chocolate.

No.	Name	TCFA mg/100g	TCA mg/100g	Manufacturer
1	Golden Label powder cacao	522	420	JSC Red October, Moscow, Russia
2	Smak powder cacao	377	1770	CJSC Presconita, Lithuania
3	Golden Anchor cacao	325	870	Bogorodskaya Confectionery Plant, LLC, Moscow, Russia
4	Alpen Gold chocolate (dark)	135		Craft Foods Rus, LLC, Pokrov, Russia
5	Luker chocolate (dark)	115		Casa Luker, Colombia
6	VAN fat free cacao	105	800	Maspex-GMV, Poland
7	Rossiysky powder cacao	97	1500	JSC Rossiya Confectionery Plant, Samara, Russia
8	Red October chocolate (bitter 80% cacao)	66		JSC Red October, Moscow, Russia
9	Vecherny Zvon chocolate	66		JSC Rot Front, Moscow, Russia
10	NOIR AUTHENTIQUE chocolate	56		FREY AG, Switzerland
11	Lux chocolate	49		JSC Babayevsky Confectionery Complex, Moscow, Russia
12	Ritter Sport chocolate with elite cacao from Ecuador (bitter 71% cacao)	47		Alfred Ritter GmbH & Co. KG, Germany
13	Black chocolate (small lumps)	45		Kakao Verarbeitung Berlin, Germany
14	Nesquik Plus cacao	45		JSC Rossiya Confectionery Plant, Samara; Nestle Russia, LLC, Russia
15	Wawel SA cacao (Naturalne cacao)	43	2570	Wawel SA, Poland
16	Alpen Gold milk chocolate with whole hazelnuts	34		Craft Foods Rus, LLC, Pokrov, Russia
17	Cacao Wedel E cacao	32	2370	Cadbury Wedel Sp, Poland
18	Hot Cocoa Mix cacao	31	139	Nestle SA, Switzerland
19	Bitter chocolate 80% cacao	27		JSC Red October, Russia
20	Coffee With Milk, chocolate milk with coffee	26		JSC Rossiya Confectionery Plant, Samara, Russia

**Table 7:** Total Content of Fat-Soluble Antioxidants (TCFA) in Nuts and Seeds (standard - gallic acid).

No.	Name	TCFA mg/100g	Manufacturer
1	Walnut	135	Russia
2	Hazelnut	99	Rosso-M, LLC, Russia
3	Pine nuts	86	Russia
4	Cashew	64	Rosso-M, LLC, Russia
5	Chestnut	63	Russia
6	Sunflower seeds	60	Rosso-M, LLC, Russia
7	Pumpkin seeds	43	Rosso-M, LLC, Russia
8	Almond	43	Rosso-M, LLC, Russia
9	Roasted peanuts	40	Rosso-M, LLC, Russia
10	Roasted hazelnuts	36	Hazelnut Promotion Group, Turkey
11	Californian almond	21	California, USA

Our databank contains TCA-related data for all kinds of tea: green, oolong, black, and puer [18]. In Table 8 are presented TCA data for green and black teas. Table 10 includes data for TCA content in coffee samples.

**Table 8:** Total Content of Antioxidants (TCA) in Green & Black Teas (standard – quercetin).

No.	Name	TCA mg/g	Manufacturer
	*Green Tea*		
1	T-sips Ceylon tea in bags	190	Sri Lanka
2	Alokozay	171	Dubai, UAE
3	Riston Green Exotic in bags	155	Sri Lanka
4	Lipton in bags	143	Unilever Foodsolutions, USA
5	Minamoto (pelleted leaves 100% of ecologically safe Japanese green tea)	143	Yunako Company, Japan
6	Nama Cha, live green tea from Japan	140	Japan
7	Tea Tang Sour Sap in bags	138	Sri Lanka
8	Azercay (Yastl cay) in bags	133	Azerbaijan
9	Greenfield flying Dragon in bags	130	London, UK
10	Merlin	127	CJSC Brand Tea, Sri Lanka
11	Gift green tea	125	Mirax Pharma
12	Nadin Super AOH Verbena	125	Vitali Tea
13	Green Elephant	125	Sri Lanka
14	Impra Blackcurrant Green Tea (rich in antioxidants)	124	Sri Lanka
15	Selenium Green Tea	124	Wahan Mingcha Tea Industry Ltd, Hubei, China
	*Black Tea*		
1	Darjeeling Tea Premium	187	Mlesna tea Naturally, bagged in Sri Lanka
2	Mabroc 1001 Nights in bags (mixture of black and green teas *etc.*)	155	Sri Lanka
3	Tea No.1 Darjeeling Rare Tea Flavor from Darjeeling	150	India
4	Darjeeling Tea	147	Djukpana Plantation, India
5	AKBAR Premium Quality Tea in bags	139	Ceylon Tea, Sri Lanka
6	Crown Gold Hilltop Collection Tea	124	English Tea Collection, Sri Lanka
7	Beta Tea Black Tea (selected quality) in bags	118	Beta Tea Groups, Turkey
8	MLESNA Presidential Ceylon Baikhovi Tea in bags	118	MLESNA, Sri Lanka
9	Tea Lisma in bags	113	May Ltd., Russia
10	Heyleys	109	Regeney Teas (PVT) Ltd., Sri Lanka
11	Bahar in bags	106	Cornill Exports (PVT) LTD, Colombo – Sri Lanka British Blend, Sri Lanka
12	Greenfield fine Darjeeling in bags	106	Greenfield Tea Ltd., UK
13	Beseda	106	Unilever Foodsolutions, Russia
14	Darjeeling	104	Chaygorod, Ltd., Russia
15	Alokozay	102	Dubai, UAE

Table 9 demonstrates TCA values for natural ground coffee from various manufacturers. The major antioxidants in coffee are aromatic oxyacids: chlorogenic acid, caffeic acid, ferulic acid, protocatechic acid *etc.* Chlorogenic acid has the highest content in coffee, and a standard 200 mL cup with 10 g of brewed coffee may contain up to 300 mL of chlorogenic acid [19,20]. The degrees of coffee roasting vary from one country to another (low, medium, strong). When brewed, the antioxidant activity of coffee decreases; this is especially true for Robusta coffee. 

**Table 9:** Total Content of Antioxidants (TCA) in Coffee (Ground) (standard – quercetin).

No.	Name	TCA mg/g
1	Maragogipe, Guatemala	32
2	Brazil	31
3	Colombia	31
4	Maragogipe Chocolate, Santo-Domingo	30
5	Ethiopia, Yirgacheffe	29
6	Puerto Rico Elite	29
7	Decaffeinated coffee	27
8	Kenya	27
9	Cuba	27
10	Jamaica elite	26
11	Sumatra	25
12	Mexico	24
13	Nicaragua	24
14	Guatemala	24
15	Honduras	24
16	Maragogipe Nicaragua	23
17	Maragogipe Mexico	23
18	Tanzania	23
19	Yemen Elite	23
20	Costa Rica	23
21	Australia Skyberry Elite	22
22	Yava	21
23	Tchibo Exclusive	14

We have measured the total contents of antioxidants (TCA) in tens of samples of red wines obtained directly from the manufacturers at elite wine expositions or purchased in Moscow stores. The wines which have been studied included ones from Chile, France, Italy, Argentina, South Africa, Macedonia, Romania, Austria, Greece, Russia, Ukraine, Georgia, and Moldova. The study showed that the total content of antioxidants in natural good wines (in reference to quercetin as a standard) varies from 250 mg/100 mL to 100 mg/100 mL (Table 10). The contents of antioxidants in some wines purchased in stores were much lower (five-fold) than the indicated values, thereby suggesting possible product adulteration. Thus, by measuring the content of antioxidants, one may obtain additional information not only about the quality or the benefits of a red wine, but also about its authenticity. According to our measures, the antioxidant activity of white wines was 5-10 times lower than that of red wines; other data showed that it was 5-20 times lower. 

**Table 10:** Total Content of Antioxidants (TCA) in Red & White Wines (standard – quercetin).

No.	Name	TCA mg/100 mL	Manufacturer
	*Red Wines *		
1	Vin de Perys D`OO	234	France
2	Selection Cabernet Saperavi 2008	220	Chateau Le Grand Vostok, Russia
3	Don Segundo	216	Agricola Kantalehos, Chile
4	Sunrise Merlo	192	Vina Concha y Toro, Chile
5	Ruby Cabernet/Pinotage	185	Winecorp PLS, RSA
6	Cabernet-Sauvignon Tamani	176	Southern Wine Company, CJSC MPBK Ochakovo, Russia
7	Cabernet	175	Bovine, Macedonia
8	Merlo	169	Bovine, Macedonia
9	Blue Pat	163	Curico Valley, Chile
10	Mukuzani	162	Tamada, Georgia
11	Merlo-Cabernet	159	Cape Town, Southern Africa
12	Merlo	159	JV Lion Gri, Moldova
	*White Wines *		
1	Stirbey	63	Provinum, Romania
2	Pino Gris	45	VJ Lion-Gri, LLC, Moldova
3	Mtsevane	36	Tamada, Georgia
4	Mtsevane	27	Old Tbilisi, Georgia
5	STOPBANKS Savignon Blanc, 2007	18	New Zealand

Cognacs contain antioxidants originating from the source cognac spirit as well as tannins and other compounds which find their way into the final product by extraction from oak casks (Table 11). Whisky also contains antioxidants by means of extraction from oak casks. We have measured the total contents of antioxidants (TCA) in cognac samples from various countries (Table 11). TCAs for many cognacs which are not specified in the Table were very low, giving reason to believe that the data was misrepresented. 

**Table 11:** Total Content of Antioxidants (TCA) in Various Cognacs, Brandy & Other Hard Liquors (standard – quercetin).

No.	Name	TCA mg/100 mL	Manufacturer
	*Brandy/Cognac*		
1	Guerin Freres (cognac)	41	Merle and Son, France
2	Vartsikhe (brandy)	33	JSC David Sarajishvili & Eniseli, Georgia
3	Rossiysky (brandy)	30	CJSC Novokubanskoye, Krasnodar Region
4	Kutuzov (brandy)	27	Moscow Inter-republic Winery, Moscow
5	Hennessy (cognac)	26	Ja Hennessy, France
6	Sarajishvili (brandy)	25	JSC David Sarajishvili & Eniseli, Georgia
7	Remi Martin V.S.O.P. (cognac)	22	France
8	Akhtamar (brandy)	22	CJSC Yerevan Cognac Distillery, Armenia
9	Janneau V.S.O.P. Armangnac, 7-year-old	19	Janneau S.A., France
10	Dacia, 25 years (brandy)	17	JSC Aroma, Moldova
11	Martell V.S.O.P. MEDAILLON (old fine cognac)	15	Martell&Co, France
12	Baku (brandy)	15	Azerbaijan
13	Kievsky, 6-year-old (brandy)	14	Moscow Wine & Cognac House KiN, Russia
	Whisky, special vodkas		
1	Ksenta Absinth	53	Torino, Italy
2	The Macallan Whisky, 12-year-old	11	Scotland
3	Bocardi Rum, 8-year-old	8	Bahamas
4	MAGONY MEZES Meggy Palinka (Cherry with honey)	7	Hungary
5	GlenfiddichWhisky	5.7	Scotland
6	Black Label Whisky	5.0	Scotland
7	Scottish Collie Whisky	4.9	Scotland
8	Chivas Regal Premium Whisky	3.6	Scotland
9	Red Label Whisky	3.0	Scotland
10	Tequila	1.6	Mexico
11	Vinogradov, Special Limon Vodka	0.04	CJSC Stolichniy Trest, Russia
12	Stolichniy Doctor Recipe No.1 No Hangover Vodka	0.03	CJSC Stolichniy Trest, Russia
13	Zelyonaya Marka Vodka	0.00	CJSC Topaz Distillery, Moscow Region, Pushkino
14	Bombay Gin	0.00	England

Quite interesting data was obtained when detecting TCA in sprouted grains (Table 12). Some of these measurements have been performed for the first time [18]. Several conclusions can be made based on the data obtained. In all cases, without exception, the amount of antioxidants in sprouted seeds increases significantly over time. When evaluating a group of grain varieties based on this parameter, it may be noted that TCA in dry seeds is not high, and the data is related to the measurements of the same order. The content of antioxidants in naked oat (Tyumensky Naked-2, author V.V. Novokhatin) is slightly higher than in wheat or rye. It may be related to the higher immunity level of this culture. On Day 5, the amount of antioxidants increases significantly in the sprouts of all three grain varieties, and, although TCA of wheat sprouts is lower than that for naked oat, the intensity of its accumulation is somewhat higher. The significant amount of antioxidants in dry buckwheat seeds, most probably due to the presence of rutin in the plant. TCA increases only twofold during the germination process; however, it reaches a certain average level. TCA in beans in higher than in cereals, particularly, in chickpea and Chickasano pea. It turned out that the 5-day sprouts of these two cultures contain very high amounts of antioxidants (503 mg/100 g and 517 mg/100 g), and this amount increases, upon germination, by 6- and 5-fold, respectively. Black sesame seeds have the highest amount of antioxidants. TCA in 5-day sprouts of this culture increases by 1.7 times when compared to dry seeds, and it reaches a significant level (490 mg/100 g). TCA in squash and flax sprouts also significantly increases, although the level of antioxidants in seeds of these cultures is not significantly higher. 

**Table 12:** Total Content of Antioxidants (TCA) in Seeds & Sprouts of Various Cultures Based on Absolutely Dry Weight (standard – quercetin).

No.	Culture	In Dry Seeds TCA mg/100 g	In Sprouts on Day 2 TCA mg/100 g	In Sprouts on Day 5 TCA mg/100 g
1	Black sesame	291	150	490
2	Saint-Mary-thistle	235	334	896
3	Buckwheat	182	203	383
4	Chickasano pea	102	263	517
5	Chickpea	84	190	503
6	Flax	56	201	526
7	Corn	42	-	-
8	Lentil	42	72	90
9	Naked oat	34	65	334
10	Squash	33	65	333
11	Rye	29	102	320
12	Zhigulevskaya Niva germinated wheat (rye) in flakes, Meta-Lux, LLC, Zhigulevsk	29	-	-
13	Wheat	24	69	275
14	Amaranth	10	17	200

Surprising results were obtained when analyzing amaranth seeds and sprouts. TCA in these seeds is only 10 mg/100 g – the lowest amount when compared to other cultures; however, by Day 5, this amount increases up to 200 mg/100 g, which is still not that high. However, the amount of antioxidants increases by 20 times during the germination process, which is twice as high when compared to the rates of accumulation of such substances for other cultures. The results related to holy thistle are of particular interest. It is known that the seeds of this wonderful plant serve as a raw material for the production of various flavonoids – in particular, quercetin, – which are to be found in several pharmacological preparations. The amount of antioxidants in dry holy thistle seeds was sufficiently high (235 mg/100 g) but lower than, let’s say, in black sesame seeds. By Day 2, it increased and was already higher than for other cultures (334 mg/100 g); by Day 5, it became significantly higher (896 mg/100 g). The experiment was extended till Day 13, and by that time, the amount of antioxidants in the holy thistle sprouts increased up to 1,000 mg/100 g, getting quite ahead of black currant. 

It should be also noted that 12 different species of plants whose sprouts were studied for the total content of water-soluble antioxidants are related to different species and varieties as well as to different families with distant phylogenic properties. It gives us reason to believe that it is common for all higher plants to increase their amounts of antioxidants with seed germination. Apart from the seeds and their sprouts, we have also compared several products obtained from grains and buckwheat seeds in terms of the content of antioxidants. These products are widely used in daily diet. For this reason, it is especially important to measure them correctly.

## 3. Experimental

### 3.1. Methods of Antioxidant Activity Measurement

For the past decades, many methods aimed at determining the level of antioxidant activity have been proposed, including new reagents, model systems and devices. Many studies related to the antioxidant activity measurement methods have been published [15,21,22,23,24,25,26,27,28]. Various chemical and physicochemical methods are applied to measure the level of antioxidant activity (AA). Such methods are primarily based on direct or indirect measurement of the reaction rate or reaction completeness. 

Three types among such methods, based on the following measurements, may be noted:

– Oxygen intake;
– Formation of oxidation products;
– Uptake (or bonding) of free radicals.

For the first and second cases, AA is determined based on the inhibition of the degree or rate of the intake of reagents or the products formation. There are several main methods for AA measurements: ORAC – oxygen radical absorbance capacity; TRAP – total radical trapping antioxidant parameter; FRAP – ferric reducing antioxidant power; TEAC (Randox) – Trolox equivalent antioxidant capacity; ABTS - [2,2-azinobis (3-ethylbenzthiazoline)-6-sulfonic acid; TBARS – thiobarbituric acid reactive substance.

In all of these methods, AA is the function of numerous parameters, including time, temperature, nature of the substance, the concentration of antioxidants and other compounds *etc*. The antioxidant activity cannot be measured directly; what is generally measured is the impact of antioxidants on the oxidation rate. All of these methods are not inter-correlated. 

The disadvantage of many antioxidant activity measurement methods is the lack of proper substrates during the measurement process. Often, the antioxidant activity towards free synthetic long-living radicals (ABTS, DPPH, AAPH *etc*.) is measured. Various synonymous terms have been proposed in the literature: “antioxidant ability”, “antioxidant power,” “antioxidant activity,” “antioxidant capacity” [26]. All of these terms are related to the antioxidant concentration (the activity of substances or substance groups). Many known methods (TEAC, TRAP, FRAP *etc.*) are based on reduction reactions of long-living free radicals or Fe (III) complex. Undoubtedly, these methods have their shortcomings as they all use synthetic free radicals, which have nothing to do with free radicals in human bodies. 

### 3.2. Amperometric Method for Measurement of Total Content of Antioxidants

The amperometric method [29] is based on measurement of electric current resulting from oxidation of the substance (or the mixture) being studied on the surface of a working electrode at a certain voltage potential. The nature of the working electrode as well as the voltage potential applied determine the sensitivity of the amperometric method. The following are used as working electrode materials: glass carbon, gold, platinum, silver, copper, nickel, palladium *etc.* The voltage potential may be set from 0 to 2.5 V. 

It has been established that the amperometric method has many advantages: low detection limit, high selectivity (only those compounds whose molecules can be oxidized are determined; other compounds which are present even in large concentrations are not determined), low cell volume (0.1-5 µL), and easy maintenance. 

Compounds containing hydroxyl groups are oxidized well under amperometric detection conditions. The major and the most active natural antioxidants are phenolic compounds. These are natural polyphenols, various flavonoids, phenolic oxyacids, vitamins, *etc.* Thus, the amperometric method is best suited for the evaluation of antioxidant activity as well as the total content of antioxidants. 

When detecting polyphenol compounds, the working electrode made of glass carbon is the most universal choice. The voltage potential may vary from 0 to 2.5 V; the ionization potential for phenol compounds varies from 100 to 1,200 mV. Electrochemical oxidation may be used as a model when measuring the activity level of free radical uptake as follows:

flavonoid-O-H→flavonoid-O**^.^** + ē + H^+^ (oxidation at a maximum voltage potential)

The antioxidant activity may be measured by using the value of oxidation of such compounds on the working electrode of the amperometric detector. The signal is registered as differential dynamic curves. Special software calculates the peak areas (of the differential curves) of the analyzed and the standard substances. The average value of 3-5 consecutive measurements are used for analysis. The following common antioxidants may be used as standard substances: rutin, quercetin, dihydroquercetin, mexidol, trolox, gallic acid, *etc.* The amperometric device has many advantages when measuring the level of antioxidant activity. Without regard to sample prep, it only takes a few minutes to carry out a measurement. The analysis (registration and processing of the results) is performed in real time; the precise dosing by a six-port valve provides for correctness and precision of the analysis; the volume of the dosing loop may vary from 20 to 500 µL; SD for valve dosing is less than 0.5%; SD for consecutive measurements for the samples being analyzed < 3%; the detection level of the amperometric detector for polyphenols, flavonoids at the level of nano-picograms (10^-9^ – 10^-12^ g). With such low amounts, the probability of a mutual impact of various antioxidants, when they are collectively present, diminishes, in particular, the manifestation of synergy. Upon detection, the electrode surface is cleaned at a high positive voltage potential for 50-200 milliseconds, then it is recovered at a negative voltage potential for 100-400 milliseconds before the new cycle begins. The operation of the amperometric detector in the pulse mode remains stable for a long time. The behavior of signals towards the same antioxidant at various voltage potentials may also be used for identification purposes. No chemical agents (save for the standards) are needed for the analysis. For this reason, the cost of the analysis is very low. The most relevant objective related to the determination of antioxidant activity is to create a universal and relatively low cost method. 

In our opinion, the amperometric technique meets such requirements more effectively than any other existing technique. The amperometric technique is especially good when comparing the antioxidant activities (AOA) of food products, various medications, beverages, and dietary supplements.

The amperometric method is the only one directly measuring the content of all antioxidants in a sample. Other methods are indirect; they measure the inhibition of reaction mixtures (particularly, free radicals), which have been generated through certain reactions [15,21,22,23,24]. The amperometric method has been successfully applied to define the antioxidant capacities of various wines [30]. This study has shown that the method is direct, precise, objective, and quick. In [31], this method was applied to determine the antioxidant capacities of olive oils obtained from various Mediterranean countries, i.e. fat-soluble samples. The method allows evaluating the quality and authenticity of olive oil. Study [32] established the antioxidant activity of lipophilic compounds in vegetables, such as carotinoids, chlorophyll, tocopherol, and capsaicine. Based on pure compounds, their antioxidant activity was evaluated (in a decreasing order): lycopene > β-carotene > zeaxanthin > α-carotene > β-crytoxanthin > lutein > α-tocopherol > capsaicine > chlorophyll α > chlorophyll β > astaxanthin > santaxanthin. The measured antioxidant activity levels of extracts from five vegetable and two fruit species were compared by using ABTS. The good correlation between the methods was obtained, save for spinach. The authors of this paper have concluded that the amperometric method can be successfully used for direct, quick, and reliable monitoring of the antioxidant capacity of lipophilic extracts of food products. The amperometric (electrochemical) method is also applied to determine the antioxidant status of a person [33]. 

The general principles of the electrochemical detection of natural antioxidants are outlined in [34]. The amperometric method, in combination with high performance liquid chromatography, is wildly used to detect polyphenols in food products and beverages [35].

We have compared results of antioxidant capacity of freeze dried products and extracts using the ORAC assay and amperometric methods (total 23 products) [17,18]. We have obtained a good correlation for these products (0.96). Such a correlation was obtained for the first time with a large amount of various products (fruits, berries, *etc.*). It is probable that the correlation could have been better if the same substance, *i.e.* Trolox, were used in both methods. In case of positive results, when comparing these methods based on more data, the amperometric method may be used along with ORAC. 

## 3. Conclusions

The original novel methodology by amperometric detection has been used to measure antioxidant capacity of different commercial available foodstuff sourced from different countries. We obtained a large volume of data about antioxidants content in foodstuffs and beverages daily used, accessible to various strata of society. All results are presented in Table 1, Table 2, Table 3, Table 4, Table 5, Table 6, Table 7, Table 8, Table 9, Table 10, Table 11 and Table 12 and allow the user to undertake a comparative analysis of antioxidant capacity of food from different sources. In many cases this can be correlated to the quality of products. This data can be used by experts of dietology, doctors for appointment of antioxidant therapy to different patients, and also to the people working in hard and harmful conditions, people who are exposed to frequent stresses, overloads (cosmonauts, miners), and to athletes before competitions, *etc.*
